# Baseline Methods for the Parameter Estimation of the Generalized Pareto Distribution

**DOI:** 10.3390/e24020178

**Published:** 2022-01-25

**Authors:** Jacinto Martín, María Isabel Parra, Mario Martínez Pizarro, Eva López Sanjuán

**Affiliations:** 1 Departamento de Matemáticas, Facultad de Ciencias, Universidad de Extremadura, 06006 Badajoz, Spain; jrmartin@unex.es (J.M.); mipa@unex.es (M.I.P.); 2 Departamento de Matemáticas, Centro Universitario de Mérida, Universidad de Extremadura, 06800 Mérida, Spain; mariomp@unex.es

**Keywords:** Bayesian inference, generalized Pareto distribution, Metropolis–Hastings algorithm, stable distributions, extreme value theory

## Abstract

In the parameter estimation of limit extreme value distributions, most employed methods only use some of the available data. Using the peaks-over-threshold method for Generalized Pareto Distribution (GPD), only the observations above a certain threshold are considered; therefore, a big amount of information is wasted. The aim of this work is to make the most of the information provided by the observations in order to improve the accuracy of Bayesian parameter estimation. We present two new Bayesian methods to estimate the parameters of the GPD, taking into account the whole data set from the baseline distribution and the existing relations between the baseline and the limit GPD parameters in order to define highly informative priors. We make a comparison between the Bayesian Metropolis–Hastings algorithm with data over the threshold and the new methods when the baseline distribution is a stable distribution, whose properties assure we can reduce the problem to study standard distributions and also allow us to propose new estimators for the parameters of the tail distribution. Specifically, three cases of stable distributions were considered: Normal, Lévy and Cauchy distributions, as main examples of the different behaviors of the tails of a distribution. Nevertheless, the methods would be applicable to many other baseline distributions through finding relations between baseline and GPD parameters via studies of simulations. To illustrate this situation, we study the application of the methods with real data of air pollution in Badajoz (Spain), whose baseline distribution fits a Gamma, and show that the baseline methods improve estimates compared to the Bayesian Metropolis–Hastings algorithm.

## 1. Introduction

Extreme value theory (EVT) is a set of statistical tools employed for modeling and predicting the occurrence of rare events outside the range of available data. It has been widely used to study events that are more extreme than any previously observed, e.g., in disciplines such as climatology: extreme events of temperature [1,2,3,4], precipitations [5,6,7,8,9,10], and solar climatology [11,12,13]; finance and insurance: applications to risk theory [14,15,16,17,18,19,20]; and engineering: design for modern buildings [21].

There are two approaches for modeling an extreme value problem. The first one is the block maxima method: dividing the sample space into blocks of equal size, the maxima values of each block follow, under a certain domain of attraction conditions, approximately a Generalized Extreme Value (GEV) distribution [22]. The second way to deal with an extreme value data set attempts to make use of the available information about the upper tail of the distribution than just the block maxima. The so-called *peaks-over-threshold* (POT) method is due to hydrologists trying to model floods. Loosely speaking, References [23,24] showed that when we consider the distribution of data above a certain threshold *u*, it can usually be approximated by a properly scaled Generalized Pareto distribution (GPD) as *u* tends to the endpoint of the distribution. This is the point of view considered in this article.

Due to its importance, several methods have been proposed for estimating the shape and scale parameters of the GPD. Classical methods include the method of moments, probability weighted moments, maximum likelihood and others. An exhaustive review of them can be consulted in [25,26]. Other researchers have proposed generalizations of the GPD: Reference [27] proposed a three-parameter Pareto distribution and employed POT to make estimations of Value at Risk and [28] introduced an extension of GPD and performed parametric estimation. However, classical methods might be inappropriate in certain situations, as explained in [26]. That is why Bayesian inference could be advisable. There are not many approaches to the GPD parameter estimation through Bayesian techniques. We can cite [29], who recommended the use of conjugate prior distributions; Reference [30] estimated the shape parameter when it is positive, and the computation of the posterior distribution was implemented via the Markov Chain Monte Carlo (MCMC) method with Gibbs sampling; Reference [31] employed Gamma and Pareto priors via MCMC; Reference [32] proposed a Bayesian mixture model, where the threshold was also a random parameter; Reference [33] employed Jeffrey’s prior and Metropolis–Hastings (MH) method and [34] employed the GPD distribution itself as the prior density.

In this paper, we aim at seizing all the available information coming from data in order to estimate the parameters of the GPD in a way as accurate as possible. A similar idea was also implemented in [35], for the estimation of the parameters of the Gumbel distribution when the baseline distribution is Gumbel, Exponential or Normal.

We will take into account all the data of the baseline distribution and study the relation between the baseline parameters and the parameters of the limit GPD in order to incorporate such relation into the sketch of new methods to make estimations. Concretely, we propose two methods and compare them with the classical MH method for data over the threshold. In addition, we will analyze four particular examples of underlying distribution: Exponential, Lévy, Cauchy and Normal, and make a special study of stable distributions.

## 2. Generalized Pareto Distribution and Its Relation with Extreme Value Theory

Let *X* be a random variable with distribution function *F*. Define *u* as the threshold value and let $X_{u}=X-u|X>u$ be the random variable with distribution function

$$F_{X_{u}}\left(x\right)=P\left[X_{u}\leq x\right]=P\left[X\leq x+u|X>u\right]=\frac{F(x+u)-F(u)}{1-F(u)},$$

for $0\leq x\leq x_{F}-u$, being $x_{F}$ the right endpoint of *F*, that is, $x_{F}:=\sup\left\{x:F\left(x\right)<1\right\}$.

Notice that $X_{u}$ is the random variable that we obtain when we consider the distribution of data above the threshold, which we usually call the tail distribution.

Given a random variable *X*, we say that it follows a Generalized Pareto Distribution (GPD) when its distribution function is

(1) $$G\left(x|\xi ,\sigma \right)=\left\{\begin{array}{cc} 1-{\left(1+\frac{\xi x}{\sigma }\right)}^{-1/\xi }, & \xi \neq 0\\ 1-\exp\left(-\frac{x}{\sigma }\right), & \xi =0 \end{array}\right.$$

where σ>0 and ξ∈R are the scale and shape parameters, respectively. Equation (1) is valid when x≥0 for ξ≥0, and for 0≤x≤−σ/ξ for ξ<0.

The fundamental result that connects EVT and GPD distribution belongs to [23,24], and it establishes that under certain mild conditions, for a random variable *X*, the distribution of $X_{u}$ for a sufficiently high threshold *u* follows a properly scaled Generalized Pareto distribution (GPD).

We will call the distribution function of *X*, *F*, the baseline distribution of the GPD. The parameters ξ and σ will depend on the value of the threshold *u*, and on the baseline distribution. For example, ξ is determined by the shape of the upper tail of the baseline distribution *F*. Positive values of the shape parameter correspond to heavy tails, while negative ones come from light tails. The special case ξ=0 will appear when the upper tail of the distribution tends to an exponential distribution of parameter 1/σ.

In this work, we will consider several types of baseline distributions for the GPD. Traditionally, estimation for the parameters of the limit GPD in Extreme Value Theory has been made taking into account only values above the threshold, but this information might be scarce. We propose a new strategy consisting in seizing all the data from the baseline distribution. We will show how this strategy can produce accurate estimations for the parameters of the GPD.

In the case when the baseline distribution is an exponential distribution with parameter λ, with distribution function $F\left(x\right)=1-e^{-\lambda x},\;x\geq 0,$ for every u≥0,

$$\begin{array}{ccc} F_{X_{u}}\left(x\right) & = & \frac{1-e^{-\lambda (x+u)}-\left(1-e^{-\lambda u}\right)}{e^{-\lambda u}}\\ & = & 1-e^{-\lambda x}=F\left(x\right),\;\forall x\geq 0. \end{array}$$

Consequently, the tail distribution $X_{u}$ is the same as the underlying distribution in the exponential case. Therefore, we must employ all the available data to estimate the parameter λ=1/σ in the definition of the GPD (1).

The case when ξ≠0 is different. In this paper, we will consider some of the most employed distributions as underlying distributions: normal distribution, which has light tails (ξ<0); and the Cauchy and Lévy distributions, which lead to heavy tails (ξ>0). Those distributions are stable; therefore, they have additional properties that will be helpful to estimate the parameters of the GPD. We will study such properties below.

With respect to the threshold, it can be settled as a known value, which has a physical meaning, depending on the characteristics of the data, or it can be defined as an upper order statistic. It is generally defined as a *p*-quantile of the underlying distribution $q_{p}$, for appropriate values of *p*.

## 3. Stable Distributions

Stable distributions are a rich class of probability distributions characterized by [36] and they have been proposed as a model for many types of physical and economic systems because of their interesting properties. They are the attractors of sums of independent, identically distributed distributions whether or not the mean or variance is finite. Good references to study them are [16] or [37].

Let *Z* be a random variable with parameters defined by its characteristic function:

(2) $$E\left[e^{itZ}\right]=\left\{\begin{array}{cc} \exp\left\{-{\left|t\right|}^{\alpha }\left(1-i\beta \tan\frac{\pi \alpha }{2}sign\left(t\right)\right)\right\}, & \mathrm{if}\;\alpha \neq 1\\ \exp\left\{-\left|t\right|\left(1+i\beta \frac{2}{\pi }sign\left(t\right)\log\left|t\right|\right)\right\}, & \mathrm{if}\;\alpha =1 \end{array}\right.$$

where the parameter α∈(0,2] is called the index of stability, and β∈[−1,1] is the skewness parameter. When β=0, the distribution is symmetric.

A random variable *X* is said to follow a stable distribution with parameters a>0 and b∈R if it satisfies that

(3) X=aZ+b.

Generally, densities can be expressed only by complicated special functions, but there are three special cases of stable distributions that have probability density functions, which can be expressed analytically:

- When α=1/2 and β=1, we obtain Lévy distributions, Z∼L(0,1). If X∼L(γ,δ), then a=δ and b=γ in (3).
- When α=1 and β=0, we obtain the family of Cauchy distributions, Z∼C(0,1). If X∼C(γ,δ), then a=δ and b=γ in (3).
- When α=2 and β=0, we obtain the normal distribution, $N(0,\sqrt{2})$. If X∼N(μ,σ), then a=σ and b=μ in (3). As usual, in this case we will denote Z∼N(0,1).

In particular, as we can standardize the stable distributions of a family, the *p*-quantiles $q_{p}$ for a stable distribution *X*, can be expressed in terms of the *p*-quantiles $z_{p}$ of the standard distribution *Z*, as

(4) $$q_{p}=az_{p}+b.$$

Let us assume that *X* follows a stable distribution with parameters *a* and *b* for Equation (3), and fix $u=q_{p}$ as the threshold for the problem of extreme value. Then, for *u* large enough, $z_{p}$ from (4) is also large, and, consequently, denoting $u_{Z}=z_{p}$, Theorem 1 guarantees that $Z_{u_{Z}}∼GPD\left(\xi_{Z},\sigma_{Z}\right)$. Therefore, as

(5) $$\frac{X_{u}}{a}=Z_{u_{Z}}$$

and

(6) $$\begin{array}{ccc} F_{X_{u}}\left(x\right) & = & P\left(X_{u}\leq x\right)=P\left(X_{u}/a\leq x/a\right)\\ & \approx & G\left(\frac{x}{a}\left|\xi_{Z},\sigma_{Z}\right.\right)=1-{\left(1+\frac{\xi_{Z}}{\sigma_{Z}}\frac{x}{a}\right)}^{-1/\xi_{Z}}, \end{array}$$

then

(7) $$X_{u}∼GPD\left(\xi_{Z},a\sigma_{Z}\right).$$

The parameter $\xi_{Z}$ will remain constant for all the random variables of the same stable family, whatever the parameters of the baseline variable are, while the scale parameter is obtained through the product of the standardization parameter *a* and the scale parameter $\sigma_{Z}$ for the GPD limiting distribution of $Z_{u_{Z}}$

(8) $$\xi =\xi_{Z},\;\sigma =a\sigma_{Z}.$$

In the case when the underlying distribution is a Cauchy or a Lévy, or any stable distribution *X* with the index 0<α<2, the tail of the distribution is considered to be “heavy", therefore it leads to a GPD where ξ>0.

Stable distributions with index of stability α≠2, (all of them except normal distribution) also verify an interesting property. As we can see in [36], given the standard distribution *Z*, its survival function $\bar{F}$ can be approximated by:

(9) $$\bar{F}\left(x\right)∼\left(1+\beta \right)C_{\alpha }x^{-\alpha },\;x\to \infty$$

where $C_{\alpha }=\frac{1}{\pi }\Gamma \left(\alpha \right)\sin\left(\frac{\alpha \pi }{2}\right)$.

From this approximation, we can infer that the shape of the tail of the distribution will only depend on the index of stability α. Therefore, if we consider the GPD that models $Z_{u_{Z}}$, the shape parameter $\xi_{Z}$ will be fixed. We will estimate it through simulation.

**Proposition** **1.** *When the baseline distribution is a standard stable distribution Z with α<2, the relation between the parameters of Z and the parameters of the GPD that models the distribution above the p-quantile of Z, $u_{Z}$, is:*

(10) $${\hat{\xi }}_{Z}=\frac{1}{\alpha },\;{\hat{\sigma }}_{Z}=\frac{1}{\alpha }{\left(\frac{C_{\alpha }\left(1+\beta \right)}{1-p}\right)}^{1/\alpha }$$

**Proof.**  From Theorem 1, for $u_{Z}$ that is big enough,

$${\bar{F}}_{u_{Z}}\left(x\right)∼{\left(1+\frac{\xi_{Z}}{\sigma_{Z}}x\right)}^{-1/\xi_{Z}}$$

and by Proposition 1 (9), also for big values of $u_{Z}$

$${\bar{F}}_{u_{Z}}\left(x\right)∼\frac{\left(1+\beta \right)C_{\alpha }x^{-\alpha }}{{\bar{F}}_{Z}\left(u_{Z}\right)}=\frac{\left(1+\beta \right)C_{\alpha }x^{-\alpha }}{1-p}.$$

Then, making equal both expressions,

$${\left(1+\frac{\xi_{Z}}{\sigma_{Z}}x\right)}^{-1/\xi_{Z}}={\left({\left[\frac{\left(1+\beta \right)C_{\alpha }}{1-p}\right]}^{-1/\alpha }x\right)}^{-\alpha }$$

Therefore, we can take ${\hat{\xi }}_{Z}=1/\alpha$ as an estimator for $\xi_{Z}$ (notice that the shape of the tail of the distribution depends only on the value of α).    □

Substituting $\xi_{Z}$ by 1/α, we have

$$1+\frac{1}{\alpha \sigma_{Z}}x={\left[\frac{\left(1+\beta \right)C_{\alpha }}{1-p}\right]}^{-1/\alpha }x,$$

so

$$1=\left({\left[\frac{\left(1+\beta \right)C_{\alpha }}{1-p}\right]}^{-1/\alpha }-\frac{1}{x}\right)\alpha \sigma_{Z}∼{\left[\frac{\left(1+\beta \right)C_{\alpha }}{1-p}\right]}^{-1/\alpha }\alpha \sigma_{Z}$$

as 1/x can be negligible. Therefore, we define

$${\hat{\sigma }}_{Z}=\frac{1}{\alpha }{\left(\frac{C_{\alpha }\left(1+\beta \right)}{1-p}\right)}^{1/\alpha }.$$

In Section 5, we will assure the accuracy of these estimators through an extensive simulation study.

## 4. Metropolis–Hastings (MH) Method

In this section, we will explain how to apply the Markov chain Monte Carlo (MCMC) method through the Metropolis–Hastings (MH) algorithm to make the estimations for stable distributions. We have to distinguish between light tails (ξ<0) and heavy tails (ξ>0). Let us assume $X_{u}∼GPD\left(\xi ,\sigma \right)$ and that we dispose of *m* values.

Let $x=(x^{1},\ldots ,x^{n})$ be a sample of *n* values from X and $x_{u}=\left(x_{u}^{1},\ldots ,x_{u}^{m}\right)$ be a sample of *m* values from $X_{u}$.

### 4.1. Light Tails ξ<0

Take k=−ξ, and $\delta =-\frac{\sigma }{\xi }$, so $X_{u}∼GPD\left(-k,k\delta \right)$, with the likelihood function

$$L\left(k,\delta |x_{u}\right)=k^{-m}\delta^{-m}\prod_{i=1}^{m}{\left(1-\frac{x_{u}^{i}}{\delta }\right)}^{-1+1/k}$$

Considering Γ($a_{0}$,$b_{0}$) and Γ($a_{1}$,$b_{1}$) as prior distributions for both parameters. Then, the MH algorithm is applied:

1. Draw a starting sample $\left(k^{\left(0\right)},\delta^{\left(0\right)}\right)$
2. For j=0,1,…
  - Sample candidates $k^{*},\delta^{*}$ from the proposal distributions
    $$k^{*}∼N\left(k^{\left(j\right)},\nu_{k}\right),\delta^{*}∼N\left(\delta^{\left(j\right)},\nu_{\delta }\right)$$
  - Calculate the ratios
    $$r_{k}=\frac{\pi (k^{*}|\delta^{\left(j\right)},x_{u})}{\pi (k^{\left(j\right)}|\delta^{\left(j\right)},x_{u})},r_{\delta }=\frac{\pi (\delta^{*}|k^{\left(j\right)},x_{u})}{\pi (\delta^{\left(j\right)}|k^{\left(j\right)},x_{u})}$$
  - Set
    $$k^{\left(j+1\right)}=\left\{\begin{array}{cc} k^{*}, & \mathrm{with}\;\mathrm{probability}\min\{1,r_{k}\}\\ k^{\left(j\right)}, & \mathrm{otherwise} \end{array}\right.$$
    $$\delta^{\left(j+1\right)}=\left\{\begin{array}{cc} \delta^{*}, & \mathrm{with}\;\mathrm{probability}\min\{1,r_{\delta }\}\\ \delta^{\left(j\right)}, & \mathrm{otherwise} \end{array}\right.$$
3. Iterate the former procedure.

Notice that

$$\begin{array}{ccc} r_{k} & = & {\left(\frac{k^{*}}{k^{\left(j\right)}}\right)}^{a_{0}-m-1}\exp\left\{b_{0}\left(k^{*}-k^{\left(j\right)}\right)+\left(\frac{1}{k^{*}}-\frac{1}{k^{\left(j\right)}}\right)\sum_{i=1}^{m}\ln\left(1-\frac{x_{u}^{i}}{\delta^{\left(j\right)}}\right)\right\}\\ r_{\delta } & = & {\left(\frac{\delta^{*}}{\delta^{\left(j\right)}}\right)}^{a_{1}-m-1}\exp\left\{b_{1}\left(\delta^{*}-\delta^{\left(j\right)}\right)+\left(\frac{1}{k^{\left(j\right)}}-1\right)\sum_{i=1}^{m}\ln\left(1-\frac{x_{u}^{i}}{\delta^{*}}\right)\right.\\ & - & \left.\left(\frac{1}{k^{\left(j\right)}}-1\right)\sum_{i=1}^{m}\ln\left(1-\frac{x_{u}^{i}}{\delta^{\left(j\right)}}\right)\right\} \end{array}$$

Finally, we obtain estimations for ξ and σ from ξ=−k and σ=kδ.

### 4.2. Heavy Tails ξ>0

In this case, the likelihood function is

$$L\left(\xi ,\sigma |x_{u}\right)=\sigma^{-m}\prod_{i=1}^{m}{\left(1+\xi \frac{x_{u}^{i}}{\sigma }\right)}^{-(1+1/\xi )}$$

Taking a type I Pareto ($a_{0},b_{0}$) as prior distribution for ξ and $\mathrm{Inv}\Gamma (a_{1},b_{1})$ for σ,

$$\begin{array}{cc} \pi (\xi ) & \propto \xi^{-(a_{0}+1)},\;\mathrm{with}\;\xi >b_{0}\\ \pi (\sigma ) & \propto \sigma^{-(a_{1}+1)}\exp\left\{-\frac{b_{1}}{\sigma }\right\} \end{array}$$

Posterior conditional distributions are

$$\begin{array}{cc} \pi (\xi |\sigma ,x_{u}) & \propto \xi^{-(a_{0}+1)}\prod_{i=1}^{m}{\left(1+\xi \frac{x_{u}^{i}}{\sigma }\right)}^{-(1+1/\xi )}\\ \pi (\sigma |\xi ,x_{u}) & \propto \sigma^{-(m+a_{1}+1)}\exp\left\{-\frac{b_{1}}{\sigma }\right\}\prod_{i=1}^{m}{\left(1+\xi \frac{x_{u}^{i}}{\sigma }\right)}^{-(1+1/\xi )} \end{array}$$

Then, MH algorithm is applied, as in the previous case.

Notice that

$$\begin{array}{cc} r_{\xi }= & {\left(\frac{\xi^{\left(j\right)}}{\xi^{*}}\right)}^{a_{0}+1}\exp\left\{\left(1+\frac{1}{\xi^{\left(j\right)}}\right)\sum_{i=1}^{m}\ln\left(1+\xi^{\left(j\right)}\frac{x_{u}^{i}}{\sigma^{\left(j\right)}}\right)-\left(1+\frac{1}{\xi^{*}}\right)\sum_{i=1}^{m}\ln\left(1+\xi^{*}\frac{x_{u}^{i}}{\sigma^{\left(j\right)}}\right)\right\}\\ r_{\sigma }= & {\left(\frac{\sigma^{\left(j\right)}}{\sigma^{*}}\right)}^{m+a_{1}+1}\exp\left\{b_{1}\left(\frac{1}{\sigma^{\left(j\right)}}-\frac{1}{\sigma^{*}}\right)+\left(1+\frac{1}{\xi^{\left(j\right)}}\right)\sum_{i=1}^{m}\ln\left(1+\xi^{\left(j\right)}\frac{x_{u}^{i}}{\sigma^{\left(j\right)}}\right)\right.\\ & -\left.\left(1+\frac{1}{\xi^{\left(j\right)}}\right)\sum_{i=1}^{m}\ln\left(1+\xi^{\left(j\right)}\frac{x_{u}^{i}}{\sigma^{*}}\right)\right\} \end{array}$$

## 5. Baseline MH Method (BMH)

In this section, we will introduce Baseline Metropolis–Hastings (BMH) method, designed according to the objectives of seizing all the available data from the baseline distribution and making use of the existing relations between the baseline parameters and the limit GPD parameters. The method consists of:

1. Applying the MH algorithm to estimate the parameters for the baseline distribution θ.
2. Making use of the relations between the baseline parameters θ and the GPD parameters ξ and σ to compute estimations for ξ and σ.

In the case of stable distributions, these relations have been explained in previous sections and are given by (8). We will detail below the application of the BMH method for the three selected stable distributions.

For the rest of the baseline distributions, the strategy to find such relations would be made thorough studies of simulation, in order to establish correspondences between the baseline parameters and the tail GPD parameters. At the moment, there are no studies in the literature about this subject; therefore, it would be interesting to perform them in future research.

Then, in the case of stable distributions, the application of BMH would be:

1. Apply the MH algorithm to estimate scale parameter *a* from the stable baseline distribution.
2. Make use of the relation (8) to compute estimations for ξ and σ, using estimators for $\xi_{Z}$ and $\sigma_{Z}$ that we detail below.

### 5.1. Estimations for $\xi_{Z}$ and $\sigma_{Z}$

In order to provide good estimations for $\xi_{Z}$ and $\sigma_{Z}$, we made a thorough simulation study for the three baseline distributions we have considered: Lévy, Cauchy, and Normal distribution. We took values for p∈[0.990,0.995] with increments of 0.001, and set the threshold $u_{Z}=q_{p}$. For each distribution, and for each value of the threshold, m=1000 values from $Z_{u_{Z}}$ were generated. This sequence was repeated 100 times. Therefore, we obtained 100 point estimations for each *p*.

**Figure 1:**
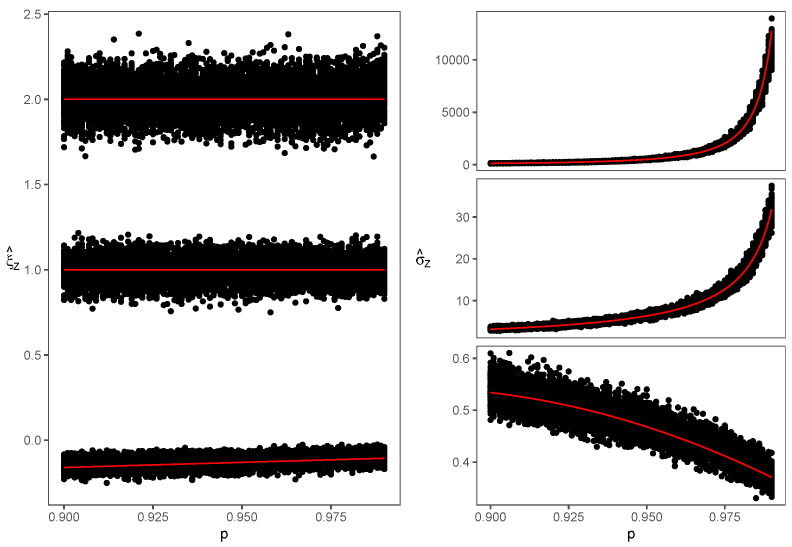
Estimations for $\xi_{Z}$ (**left**) and $\sigma_{Z}$ (**right**) for Lévy (**upper charts**), Cauchy (**middle charts**), Normal (**lower charts**) and estimators from Equations (11)–(13) plotted in red for p∈[0.900,0.995].

To guarantee the convergence of the MCMC algorithm, we must be sure that the posterior distribution has been reached. These proceedings were made using library coda [38] for R software, taking 10,000 values for the burn-in period, 25 values for the thinning, and selecting initial values for each sample. Finally, to obtain the posterior distribution for each parameter, a Markov chain of length 10,000 was obtained and we considered the mean as the estimator. The results of the simulation study are shown in Figure 1.

#### 5.1.1. Lévy and Cauchy Baseline Distribution

By Proposition 1 (10), we had estimations for $\xi_{Z}$ and $\sigma_{Z}$. Concretely, these are

(11) $${\hat{\xi }}_{Z}=2,\;{\hat{\sigma }}_{Z}=\frac{4}{\pi }{\left(1-p\right)}^{-2},$$

for the Lévy distribution and,

(12) $${\hat{\xi }}_{Z}=1,\;{\hat{\sigma }}_{Z}=\frac{1}{\pi }{\left(1-p\right)}^{-1}$$

for the Cauchy distribution.

#### 5.1.2. Normal Baseline Distribution

In the case of the normal distribution, property (10) is not verified. In this case, the adjustment from the simulation study is:

(13) $${\hat{\xi }}_{Z}=-0.7+0.61p,\;{\hat{\sigma }}_{Z}=0.34+3.18\left(1-p\right)-12.4{\left(1-p\right)}^{2}.$$

Now, we will estimate the scale parameter *a* of the baseline distribution *X*. Notice that parameter *b* for the stable distribution *X* defined in (3) does not have any influence on the estimation of the parameters of the GPD, as we have shown before. Consequently, we will assume b=0 from now on.

### 5.2. Lévy Distribution

Likelihood function for Lévy distribution is:

$$L\left(a|x\right)\propto a^{n/2}\exp\left\{-\frac{a}{2}\sum_{i=1}^{n}\frac{1}{x^{i}}\right\}$$

Taking a prior distribution $\Gamma (a_{0},b_{0})$ for *a*, and making use of (8) and (11), we obtain estimations $\hat{\xi }$ and $\hat{\sigma }$.

### 5.3. Cauchy Distribution

Likelihood function for Cauchy is:

$$L\left(a|x\right)\propto a^{-n}\prod_{i=1}^{n}{\left(1+{\left(\frac{x^{i}}{a}\right)}^{2}\right)}^{-1}$$

Taking $\Gamma (a_{0},b_{0})$ as prior distribution and, making use of (8) and (12), we obtain estimations $\hat{\xi }$ and $\hat{\sigma }$.

### 5.4. Normal Distribution

In this case, we consider $\mathrm{Inv}\Gamma (a_{0},b_{0})$ as prior distribution for $a^{2}$, and making use of (8) and (13), we obtain estimations $\hat{\xi }$ and $\hat{\sigma }$.

## 6. Informative Priors Baseline MH (IPBMH)

Finally, we propose an MH method to estimate the parameters ξ and σ, where highly informative priors are employed, seizing the estimations computed before. Employing estimations of $\xi_{Z}$, $\sigma_{Z}$ and *a* obtained in previous sections, we can settle priors for ξ and σ, which are very informative. Notice that this way of proceeding keeps the original idea of Extreme Value Theory, granting more weight to tail values because they are employed twice: to compute estimations for $\xi_{Z}$ and $\sigma_{Z}$ and also through the likelihood function. As we commented before, this method could also be implemented for other baseline distributions once we have found relations between baseline and GPD parameters.

For stable distributions, highly informative priors are

$$\begin{array}{c} \xi ∼N\left(\xi_{Z},b_{1}\right),\sigma ∼N\left(a\cdot \sigma_{Z},b_{2}\right) \end{array}$$

Then, for the three distributions studied, *a* is estimated through BMH and $\xi_{Z}$, $\sigma_{Z}$ are estimations computed through (11)–(13). In addition,

- $b_{1}$ is constant, being 0.03, 0.065 and 0.1 for Normal, Cauchy and Lévy baseline distributions, respectively.
- $b_{2}=\exp\left\{c_{1}p^{2}+c_{2}p+c_{3}\right\}$, where values are given in Table 1.

The Joint posterior distribution is

$$\begin{array}{cc} \pi (\xi ,\sigma |x_{u}) & \propto \sigma^{-m}\exp\left\{-\frac{1}{2b_{1}^{2}}{\left(\xi -\xi_{Z}\right)}^{2}-\frac{1}{2b_{2}^{2}}{\left(\sigma -a\cdot \sigma_{Z}\right)}^{2}\right\}\prod_{i=1}^{m}{\left(1+\xi \frac{x_{u}^{i}}{\sigma }\right)}^{-(1+1/\xi )} \end{array}$$

and marginal distributions are

$$\begin{array}{cc} \pi (\xi |\sigma ,x_{u}) & \propto \exp\left\{-\frac{1}{2b_{1}^{2}}{\left(\xi -\xi_{Z}\right)}^{2}\right\}\prod_{i=1}^{m}{\left(1+\xi \frac{x_{u}^{i}}{\sigma }\right)}^{-(1+1/\xi )}\\ \pi (\sigma |\xi ,x_{u}) & \propto \sigma^{-m}\exp\left\{-\frac{1}{2b_{2}^{2}}{\left(\sigma -a\cdot \sigma_{Z}\right)}^{2}\right\}\prod_{i=1}^{m}{\left(1+\xi \frac{x_{u}^{i}}{\sigma }\right)}^{-(1+1/\xi )} \end{array}$$

Then, we apply the MH algorithm with

$$\begin{array}{cc} r_{\xi } & =\exp\left\{\frac{1}{2b_{1}^{2}}\left({\left(\xi^{\left(j\right)}-\xi_{Z}\right)}^{2}-{\left(\xi^{*}-\xi_{Z}\right)}^{2}\right)\right.\\ & \left.-\left(1+\frac{1}{\xi^{*}}\right)\sum_{i=1}^{m}\ln\left(1+\xi^{*}\frac{x_{u}^{i}}{\sigma^{\left(j\right)}}\right)+\left(1+\frac{1}{\xi^{\left(j\right)}}\right)\sum_{i=1}^{m}\ln\left(1+\xi^{\left(j\right)}\frac{x_{u}^{i}}{\sigma^{\left(j\right)}}\right)\right\} \end{array}$$

$$\begin{array}{cc} r_{\sigma } & ={\left(\frac{\sigma^{\left(j\right)}}{\sigma^{*}}\right)}^{m}\exp\left\{\frac{1}{2b_{2}^{2}}\left({\left(\sigma^{\left(j\right)}-a\cdot \sigma_{Z}\right)}^{2}-{\left(\sigma^{*}-a\cdot \sigma_{Z}\right)}^{2}\right)\right.\\ & \left.-\left(1+\frac{1}{\xi^{\left(j\right)}}\right)\sum_{i=1}^{m}\ln\left(1+\xi^{\left(j\right)}\frac{x_{u}^{i}}{\sigma^{*}}\right)+\left(1+\frac{1}{\xi^{\left(j\right)}}\right)\sum_{i=1}^{m}\ln\left(1+\xi^{\left(j\right)}\frac{x_{u}^{i}}{\sigma^{\left(j\right)}}\right)\right\} \end{array}$$

## 7. Simulation Study

Now, we will develop a thorough simulation study in order to compare the accuracy of the three MH methods of estimation: MH, BMH and IPBMH.

We fixed p=0.9 and the threshold $u=q_{p}$. Furthermore, we take b=0. We sample $n=2^{i}$, with i=5,…,10 values from the three baseline distributions considered, with scale $a=2^{j},j=-2,-1,0,1,2$. We obtained an MCMC with length 10,000, taking 10,000 values for the burn-in period, 25 values for the thinning and selecting initial values for each sample. Finally, this sequence was repeated 100 times and we considered the mean as the estimator.

**Figure 2:**
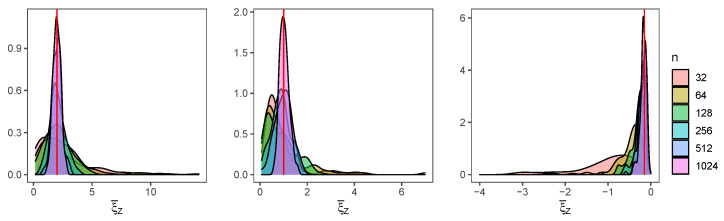
Posterior distribution of $\xi_{Z}$ for the different values of *n*, when the baseline distribution is L(0,1) (**left**), C(0,1) (**middle**) and N(0,1) (**right**), for the MH method. The estimation of BMH is plotted as a vertical red line.

In Figure 2, we can see the posterior distribution of the parameters $\xi_{Z}$ and $\sigma_{Z}$ for the different sample sizes *n*, when the baseline distribution is L(0,1) (left), C(0,1) (middle) and N(0,1) (right), for the methods MH and BMH. For the first one, the distribution is right skewed, although skewness becomes smaller as *n* increases. BMH offers a point estimation, plotted as a vertical red line.

**Figure 3:**
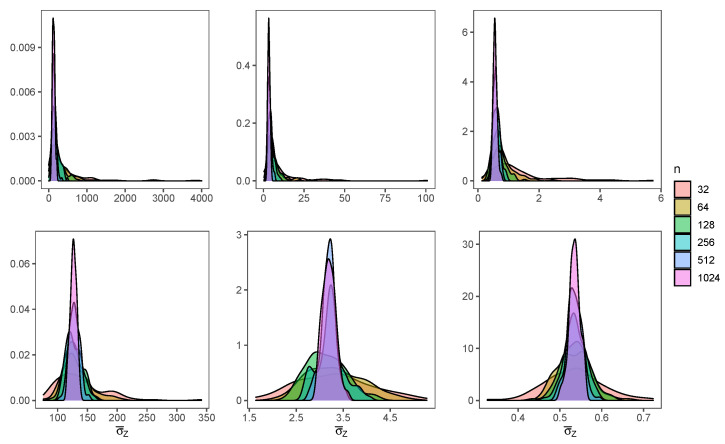
Posterior distribution of $\sigma_{Z}$ for the different values of *n*, when the baseline distribution is L(0,1) (**left**), C(0,1) (**middle**) and N(0,1) (**right**), for the method MH (**upper charts**) and BMH (**lower charts**).

In Figure 3, we can see the posterior distribution for $\sigma_{Z}$, for both methods. MH (upper charts) shows much more skewness, such as in the case of $\xi_{Z}$, while estimations from BMH (lower charts) are less skewed and dispersed.

**Figure 4:**
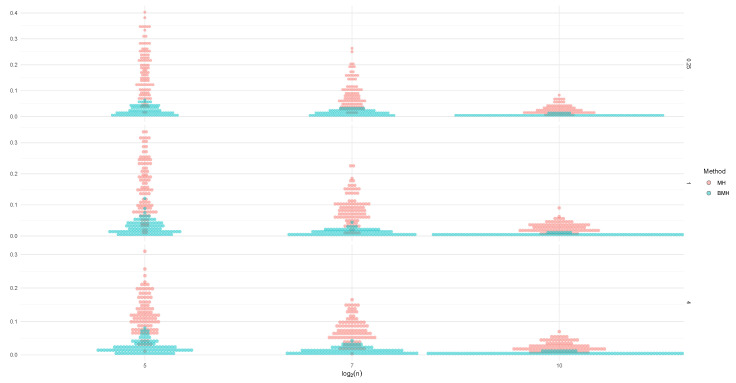
MAE for MH and BMH when a=0.25,1,4, and for sample sizes $n=2^{5},2^{7},2^{9}$, for the baseline distribution L(0,1).

**Figure 5:**
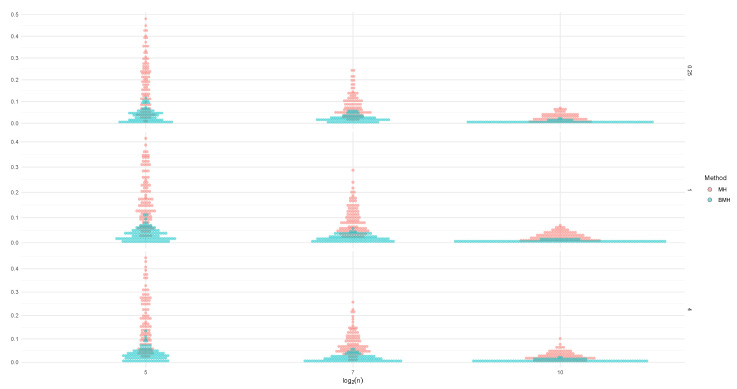
MAE for MH and BMH when a=0.25,1,4, and for sample sizes $n=2^{5},2^{7},2^{9}$, for the baseline distribution C(0,1).

**Figure 6:**
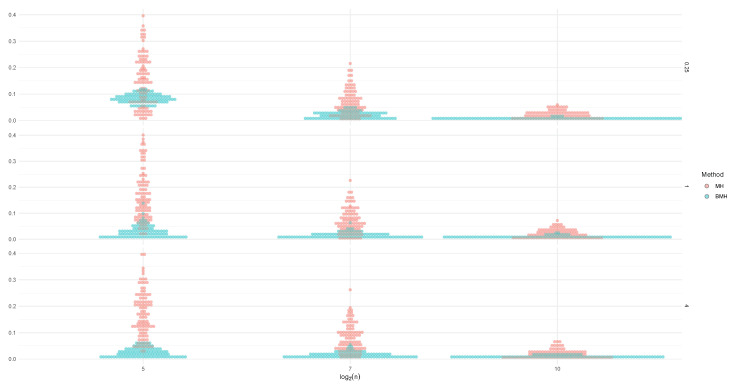
MAE for MH and BMH when a=0.25,1,4, and for sample sizes $n=2^{5},2^{7},2^{9}$, for the baseline distribution N(0,1).

Now, we will compare mean absolute errors (MAE) for MH and BMH when a=0.25,1,4, and for sample sizes $n=2^{5},2^{7},2^{9}$. In Figure 4, Figure 5 and Figure 6, we can see how BMH provides smaller errors than MH.

Clearly, BMH provides more accurate estimations for the parameters of the GPD when the baseline distribution is stable and known. However, in practical situations, we might not know which is the baseline distribution, or data could better fit a mixture of distributions rather than a simple one. In these situations, the use of highly informative priors, built with the information available from all the data, could be advisable. To develop this idea, we simulated from different mixtures and computed values of MAE for the three methods, finding that IPBMH is the method that shows the best behavior when data differ from the simple distributions.

**Figure 7:**
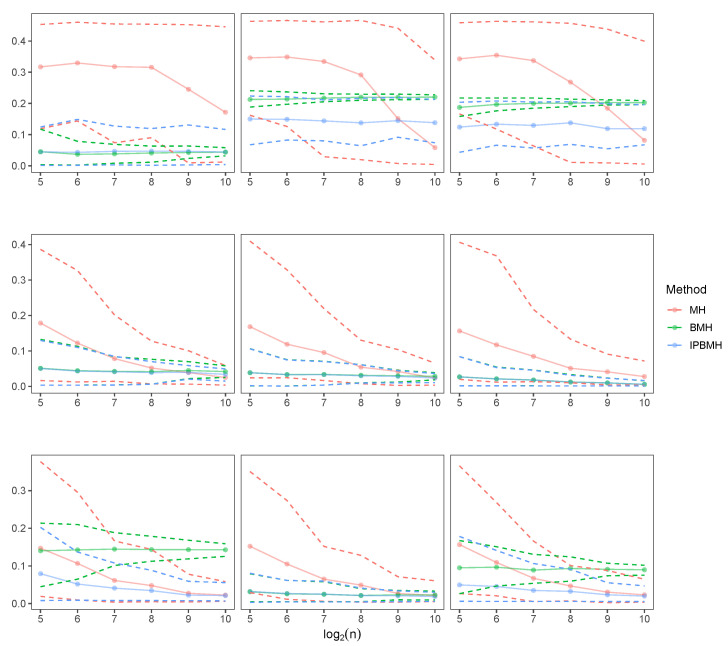
MAE and 2.5%, 97.5% quantiles for the three methods, for αF(0,1/2)+(1−α)F(0,2) (**left charts**), αF(0,1)+(1−α)F(1,1) (**middle charts**) and αF(0,1)+(1−α)F(1,2) (**right charts**), for Lévy (**upper charts**), Cauchy (**medium charts**), Normal (**lower charts**).

In Figure 7, we can see MAE for the three methods, in the case of the mixtures employed (α=0.5): αF(0,1/2)+(1−α)F(0,2) (left charts), αF(0,1)+(1−α)F(1,1) (middle charts) and αF(0,1)+(1−α)F(1,2) (right charts), for the three baseline distributions considered (Lévy, Cauchy, Normal). In general, IPBMH is the most advisable method, especially for the case when data are scarce. Notice that when data approaches one of the pure stable distributions, for example, in the case of Cauchy mixtures (which are still quite similar to a simple Cauchy) and the second mixture for the Normal distribution, BMH and IPBMH show very similar results. However, when the mixtures differ significantly from the simple distribution, the method IPBMH and MH are more advisable than BMH.

## 8. An Application: PM 2.5 in Badajoz (Spain) during the Period 2011–2020

As we mentioned before, both baseline methods can be generalized for other baseline distributions by studying the relations between the parameters of the baseline distributions and the parameters of the limit GPD. We show an example, employing real data whose baseline distribution can be fitted by a Gamma distribution.

**Figure 8:**
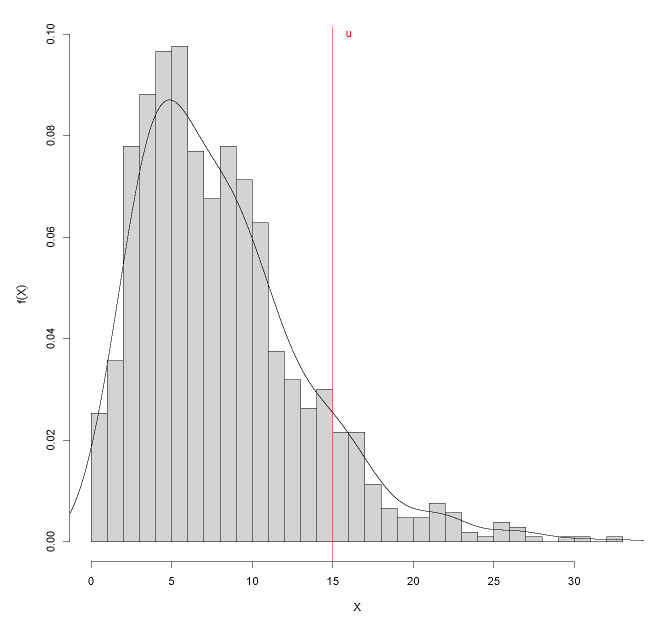
Histogram of PM 2.5 (in μg/m^3^) in Badajoz for 2011–2020, threshold $u=q_{p}=15$, p=0.90 and density (black curve).

Data from measurements of the levels for diverse air pollutants in many municipalities in Spain are publicly available on the website https://www.miteco.gob.es/es/calidad-y-evaluacion-ambiental/temas/atmosfera-y-calidad-del-aire/, accessed on 15 January 2022. Particulate matter is a mixture of solid particles and liquid droplets that can be inhaled and cause serious health problems. In particular, we have selected particulate matter less than 2.5 micrometers in diameter, known as PM 2.5, because it is considered to be especially dangerous to human health. In this context, studying the tail distribution above a certain threshold is essential because the World Health Organization recommends not to exceed an average daily value of 25 μg/m^3^ and not to exceed an average annual value of 10 μg/m^3^. We studied levels of PM 2.5 measured in μg/m^3^ for the last ten years available, 2011–2020, from Badajoz. There are n=1066 observations, and, as can be seen in Figure 8, data can be fitted by a Gamma distribution. As in the previous simulations, p=0.90 and the threshold $u=q_{p}$, resulting u=15 μg/m^3^.

Making a simulation study, similar to the previous ones made for Normal, Lévy and Cauchy, we obtained the following relation between the parameters of the baseline distribution Γ(α,β) and the parameters of limit GPD (ξ,σ):

(14) $$\hat{\xi }=0,\;\hat{\sigma }=\frac{1}{\beta }\left(1+0.22\log_{2}\alpha \right)$$

**Figure 9:**
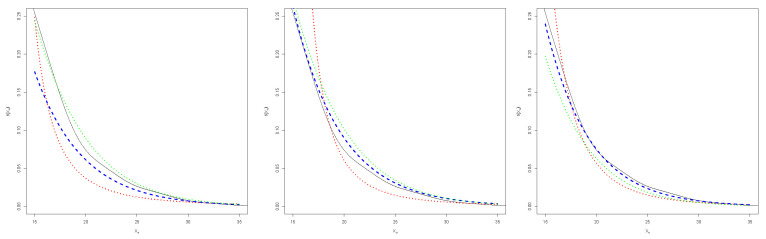
Tail distribution of data, density (black curve), estimations for MHM (red colored), BDM (green colored) and IPBDM (blue colored).

Then, we randomly selected 50 data and applied the three methods MHM, BDM and IPBDM to fit the tail data. This proceeding was repeated many times, and we found three possible behaviors, as shown in Figure 9. In the first case (left chart), there is an example of the most usual situation: IPBDM offers intermediate estimations, between MHM and BDM. When there are scarce tail data (middle chart), MH differs significantly from the real density, while BDM and IPBDM provide better estimations. Finally, in the right chart, a common situation is shown in which IPBDM clearly offers the best estimations.

## 9. Conclusions

1. In the parameter estimation of GPD, usual EVT methods waste a lot of information. We have proposed two MH methods that make the most of all the information available from the data set. They are based on making use of the existing relations between baseline and GPD parameters, through informative priors.
2. When considering the GPD coming from stable baseline distributions, we employed singular properties of stable distributions to simplify the problem (reducing to standard cases) and to provide estimators for the parameters of the GPD.
3. We have achieved very accurate estimations for the parameters of the GPD when the baseline distribution is Cauchy, Normal or Lévy, making use of the properties of stable distributions and MH methods.
4. We have studied the goodness of the estimations for classical MH method and BMH when the baseline distribution is standard Cauchy, Normal or Lévy. Clearly, BMH provides more accurate estimations than MH.
5. In most real situations, data do not fit a simple distribution. We simulated some examples of mixtures of stable distributions and showed that IPBMH provides more accurate estimations than the other methods.
6. These proposals could be generalized for other baseline distributions by studying the relations between the parameters of the baseline distributions and the parameters of the limit GPD. We provide an application with real data to illustrate this situation.

## Figures and Tables

**Table 1:** Values for $c_{1}$, $c_{2}$, $c_{3}$ for the three baseline distributions.

Distribution	$c_{1}$	$c_{2}$	$c_{3}$
Lévy	500.2	−900.9	408.2
Cauchy	323.57	−588.51	266.13
Normal	−46.24	83.55	−41.58

## Data Availability

Not applicable.
